# The Diversity in Tobacco Use Among Women of Reproductive Age (15–49 Years) in Pakistan: A Secondary Analysis of a Multiple Indicator Cluster Survey 2016–2018

**DOI:** 10.1093/ntr/ntae016

**Published:** 2024-02-01

**Authors:** Radha Sharma, Mona Kanaan, Kamran Siddiqi

**Affiliations:** Department of Health Sciences, University of York, York, UK; ConnectHEOR Canada Limited, Edmonton, Alberta, Canada; Department of Health Sciences, University of York, York, UK; Department of Health Sciences, University of York, York, UK; Hull York Medical School, University of York, York, UK

## Abstract

**Introduction:**

Tobacco use in women of reproductive age group (15–9 years) may contribute to poor maternal and child health outcomes. We estimated the prevalence of smokeless tobacco (ST), cigarette, and waterpipe use among these women in Pakistan and explored associations with socio-economic differences and birth weights after recent childbirths (past 2 years).

**Aims and Methods:**

We used the Multiple Indicator Cluster Surveys datasets from three provinces in Pakistan. Prevalence was generated for current use of tobacco among women with recent childbirth (WWRC) and without recent childbirth (WWoRC). We further compared socio-economic status and birth weights between tobacco users and non-users.

**Results:**

The prevalence of current ST use was 1.4% and 2.5%, and waterpipe smoking was 1.2% and 1.8%, in WWRC and WWoRC, respectively. ST use varied across Pakistan’s provinces by 13 percentage points in WWRC and WWoRC while waterpipe smoking by 10 and 15 percentage points, respectively. The odds of using any form of tobacco were significantly lower with higher levels of education or wealth index. Compared to children born to mothers who did not use tobacco, the birth weight of children born to mothers who smoked waterpipe was on average 0.83 kg (95% CI −1.6 to −0.1) lower whereas it was 0.33 kg (95% CI −0.9 to 0.3) higher for ST users.

**Conclusions:**

Tobacco use among women of reproductive age in Pakistan varied significantly based on province and tobacco type. Waterpipe smoking was associated with a reduction in birth weight. Raising awareness of the harms of tobacco use among women of reproductive age and targeting interventions in high-burden regions of Pakistan should be prioritized.

**Implications:**

The prevalence of current smokeless tobacco use and waterpipe smoking varied significantly in Pakistan (~13 and ~15 percentage points, respectively) among women of reproductive age, and there seems to be little change in tobacco use behavior around pregnancy and early motherhood. The study highlights a need to better understand the prevalence of tobacco use in Pakistan, and further contextual research is warranted to understand the reasons for such high tobacco use in certain areas. This information highlights the need for improving current tobacco control interventions and policies, including measures that could help reduce its use and prevent its uptake. Furthermore, compared to children born to mothers who did not use any form of tobacco, we found a statistically significant difference (830 g) in birth weight for babies born to mothers who at the time of the interview smoked waterpipe and a non-statistically significant difference of about 330 g for children born to mothers who at the time of the interview used smokeless tobacco. In addition to our findings, some literature suggests the association of waterpipe smoking with low birth weight. A possible explanation could be the exposure to smoke that contains toxicants from the tobacco product itself in addition to the burning of charcoal, which is required when using waterpipe. Waterpipe smoking also results in high exposures to carbon monoxide and polycyclic aromatic hydrocarbons, which are known to be carcinogenic and teratogenic. In terms of smokeless tobacco use, further research is warranted to understand its impact on birth outcomes through longitudinal studies accounting for diverse products, its constituents and the amount of consumption.

## Introduction

Tobacco use among women of reproductive age affects several perinatal and reproductive outcomes.^1–4^ This is in addition to several other harms of tobacco, such as cardiovascular diseases and cancers.^5,6^ Most tobacco risk estimates originate from high-income countries (HIC) and are based on cigarette smoking. Yet in many low- and middle-income countries (LMICs), smokeless tobacco use and waterpipe smoking are equally or more popular, particularly among women.^7,8^ Waterpipe smoking is also gaining worldwide popularity among youth.^9,10^

In South Asia, tobacco use is particularly diverse, with most women using smokeless tobacco and waterpipe—also known as hookah/sheesha/nargileh.^11–13^ The age- and sex-specific standardized prevalence of smokeless tobacco among adults aged 15 and older, spanning 204 countries, indicated that approximately 273.9 million people globally used smokeless tobacco in 2019. The majority of these users, about 228 million (83%), were located in the South Asia region, with the highest prevalence observed among females in South Asia (12%).^14^ While cigarette smoking rates remain low in South Asia, other tobacco products are highly prevalent among women due to their accessibility and social acceptability.^12,15^ Social factors such as education, income, perceived health benefits, social acceptability, and peer habits determine its intake.^16,17^ Furthermore, this high prevalence also extends to its use during pregnancy and breastfeeding.^12,18^

In Pakistan, the overall prevalence of smokeless tobacco use, waterpipe smoking, and cigarette smoking among women is 3.7%, 1.1%, and 1.0%, respectively.^19^ However, consumption rates vary; for example, within urban settlements in Karachi, smokeless tobacco prevalence among women can be as high as 42%,^20^ while another study reported the prevalence of waterpipe smoking as 41%.^21^ This indicates that consumption is likely to be determined by socio-ecological factors, which are currently unreported in the literature. A good understanding of these socio-ecological factors is crucial for any future research and policy work to address tobacco use in women of reproductive age group in Pakistan.

Tobacco use in any form is harmful to human health. While, the causal link between cigarette smoking and adverse health effects is well-established, the evidence on the harms of smokeless tobacco and waterpipe smoking is also strong and emerging. Smokeless tobacco is associated with oral and upper aerodigestive tract cancers and cardiovascular disease-related deaths.^22–25^ Waterpipe smoking is associated with chronic obstructive pulmonary diseases, several malignancies, and cardiovascular diseases.^26–29^ Despite the notable harms of smokeless tobacco and waterpipe smoking and its high prevalence in certain populations, there is limited literature on their effect on perinatal outcomes.^30^

There is a dearth of literature on the prevalence, associations and effects of smokeless tobacco, and waterpipe use among women of reproductive age. Furthermore, recent tobacco use estimates, especially based on the different types of tobacco are not available. These estimates are required to understand the current tobacco-related burden among women of reproductive age. This study aimed to understand the environmental and socio-economic reasons for the variation in prevalence estimates in Pakistan and the association between tobacco use and birth weight. The findings from this study will inform policy and contribute toward intervention development to address tobacco use among women of the reproductive age group. Furthermore, understanding the fundamentals of tobacco use among these women will enable tailored preventive measures to be implemented to avoid the uptake of tobacco in the first place.

## Methods

### Data

We analyzed cross-sectional survey data from the Multiple Indicator Cluster Surveys (MICS) VI datasets (most recent) from Sindh (2018), Khyber–Pakhtunkhwa (KP) (2016/17) and Punjab (2017) provinces of Pakistan. The (freely available) MICS data is collected by the United Nations International Children’s Emergency Fund (UNICEF). It is a large, representative dataset on maternal and child health collected by standardized methods in each province, by first identifying the rural and urban areas in all districts as main sampling strata, then systematically selecting a specified number of census enumeration areas within each stratum, and finally selecting a systematic sample of 20 households in each enumeration area.^31^

### Measures

The questions asked during the survey and responses for the variables used in this study are mentioned below:

#### Tobacco Use

Women were asked if they ever tried or used ST, smoked cigarettes, and smoked other non-cigarette forms of tobacco (referred as waterpipe smoking in this paper), which were reported as a binary variable (yes/no). Women who reported ever use were further asked about their tobacco use within the past month (binary variable: yes/no) referred as current tobacco use in this paper. In this paper, we are focusing on the current use of tobacco.

#### Birth Weight

All women were asked if they had a live birth in the past 2 years as of the date of the interview (yes/no), and those who responded yes, were further asked additional questions on maternal and child history, including birth outcomes. Birth weight data were collected by asking women whether the child was weighed at birth (yes/no). If the answer was yes, they were further asked if they had the child’s weight recorded in the delivery record card. Based on that, birth weight was reported in kilograms specifying whether the value originated from the delivery record card or by recall.

#### Sociodemographic Covariates

Age (in completed years at the time of the interview) recorded as a continuous value, highest education attained (primary, middle, secondary, and higher), area of residence (rural or urban), ecological data (ie, the divisions and sub-divisions in each province), and quintiles of wealth index (poorest, second, middle, fourth, and richest) were reported. Age and education were individual indicators at the mother’s level, while the area of residence, ecological data and wealth index were part of household indicators. The wealth index was constructed using principal components analysis,^32,33^ based on various characteristics related to a household’s material wealth.^31^

## Statistical Analysis

The secondary data analysis was performed in STATA (StatsCorp, 2017). Descriptive summary statistics for the characteristics (age, education, and wealth index) of all women interviewed (15–49 years) who reported birth history in the previous 2 years (women with and without live birth in the previous 2 years) were calculated by provinces and their divisions. Prevalence estimates for current smokeless tobacco use, cigarette smoking, and waterpipe smoking, and 95% confidence intervals (CI), were estimated and further stratified by divisions in each province. Pooled estimates for each province and for the country were also generated. Sampling weights were accounted for differential probabilities of selection and participation in the calculation of summary statistics and prevalence estimates. Sampling weights were calculated using the *svy* command in STATA based on the weight variables provided in the MICS dataset.

Regression analyses were conducted by combining the data from all divisions after accounting for the possible clustering effect by division. The following analyses were performed:

Logistic regression was used to explore the effect of maternal covariates (namely, residence, education, socio-economic status, and age) on current use of tobacco for each of the following tobacco forms: ST, cigarette, and waterpipe smoking. In each case, non-tobacco users formed the reference group.

Multiple linear regression analysis to estimate the effect of current tobacco use on birth weight (outcome variable reported as a continuous variable in kilograms) among women who have had a live birth in the past 2 years, compared to non-tobacco users. The independent variables were current exclusive smokeless tobacco use, exclusive cigarette smoking, and exclusive waterpipe smoking (dual users were not considered due to extremely small observations). For this particular analysis, we created a new variable with exclusive use of any form of tobacco to estimate their effect on birth weight compared to non-tobacco users.

For all regression analyses, we reported 95% CIs for the estimates. A *p*-value less than .05 was considered statistically significant.

## Results

The study included data of 85 412 women of reproductive age: 31 210 women with and 54 202 women without a live birth in the past 2 years. Furthermore, birth weight data were only available for 15.4% (*n* = 4790) of women with recent childbirth. The mean ages of women with and without recent childbirth were 28.3 and 36 years, respectively. About 70% (women with recent childbirth) and 63% (women without recent childbirth) lived in rural areas; more than half (52% and 57% among women with and without a recent childbirth, respectively) had no formal education. Wealth index distribution was similar among women with and without a recent childbirth. Detailed characteristics of these women are reported in Tables 1 and 2.

**Table 1.** Socio-Demographic Distribution (Weighted) of Women With a Live Birth in the Past 2 Years (15-49 Years)

Socio-demographic distribution (women WITH one or more live births in the previous 2 years)														
		Women (weighted)	Mean Age (years)	Rural dwellers *n* (%)	Education *n* (%)					Combined Wealth Index *n* (%)				
Province	Division				None/preschool	Primary	Middle	Secondary	Higher	Poorest	Second	Middle	Fourth	Richest
Punjab	Bhawalpur	1560	28.69	1219 (78.17)	899 (32.29)	521 (18.66)	344 (12.34)	408 (14.62)	615 (22.08)	637 (40.86)	361 (23.12)	282 (18.06)	172 (11.06)	108 (6.9)
	DG Khan	1746	28.37	1487 (85.17)	1150 (65.88)	308 (17.64)	104 (5.97)	100 (5.74)	83 (4.77)	1037 (59.43)	416 (23.84)	173 (9.90)	79 (4.55)	40 (2.28)
	Faisalabad	1902	28.59	1248 (65.63)	702 (36.89)	421 (22.15)	205 (10.79)	285 (15)	288 (15.16)	372 (19.58)	392 (20.63)	394 (20.70)	407 (21.41)	336 (17.68)
	Gujranwala	2236	29.21	1448 (64.77)	484 (21.63)	448 (20.04)	350 (15.66)	469 (20.98)	485 (21.70)	89 (3.99)	243 (10.85)	542 (24.23)	739 (33.06)	623 (27.88)
	Lahore	2623	28.33	905 (34.52)	838 (31.94)	438 (16.69)	332 (12.64)	442 (16.86)	574 (21.87)	177 (6.76)	360 (13.72)	495 (18.88)	675 (25.73)	916 (34.91)
	Multan	1852	28.05	1374 (74.21)	866 (46.73)	406 (21.93)	188 (10.14)	210 (11.33)	183 (9.88)	446 (24.07)	515 (27.83)	423 (22.84)	270 (14.56)	198 (10.71)
	Rawalpindi	1341	29.17	843 (62.9)	321 (23.91)	258 (19.22)	170 (12.65)	301 (22.47)	292 (21.76)	61 (4.53)	157 (11.73)	306 (22.82)	367 (27.34)	450 (33.58)
	Sahiwal	1100	28.88	866 (78.68)	516 (46.91)	261 (23.71)	102 (9.31)	120 (10.95)	100 (9.13)	255 (23.17)	337 (30.66)	281 (25.54)	143 (13)	84 (7.62)
	Sargodha	1296	28.71	1007 (77.73)	592 (45.64)	301 (23.23)	107 (8.29)	164 (12.68)	132 (10.15)	358 (27.63)	329 (25.35)	287 (22.12)	228 (17.59)	95 (7.30)
	**Provincial**	**15 656**	**28.64**	**10 399 (66.42)**	**6365 (40.66)**	**3126 (19.97)**	**1663 (10.62)**	**2248 (14.36)**	**2254 (14.39)**	**3433 (21.93)**	**3110 (19.87)**	**3182 (20.32)**	**3080 (19.68)**	**2850 (18.20)**
Sindh	Hyderabad	1389	29.1	952 (68.54)	1030 (74.20)	142 (10.21)	46 (3.29)	65 (4.66)	106 (7.65)	530 (38.20)	341 (24.55)	242 (17.44)	119 (8.58)	156 (11.23)
	Karachi	1523	28.15	130 (8.53)	494 (32.40)	175 (11.52)	197 (12.94)	322 (21.17)	334 (21.96)	11 (0.75)	24 (1.56)	200 (13.14)	681 (44.71)	607 (39.84)
	Larkana	1003	29.04	703 (70.09)	782 (78.01)	106 (10.62)	25 (2.49)	29 (2.86)	60 (6.01)	242 (24.12)	432 (43.12)	221 (22.03)	79 (7.88)	28 (2.85)
	Mirpur Khas	654	28.22	519 (79.38)	491 (75.20)	62 (9.41)	18 (2.76)	45 (6.89)	37 (5.74)	380 (58.21)	113 (17.31)	86 (13.23)	43 (6.58)	30.53 (4.67)
	Shaheed Benazirabad	681	29.52	492 (72.16)	496 (72.78)	87 (12.61)	32 (4.71)	37 (5.39)	31 (4.51)	141 (20.66)	269 (39.49)	178 (26.08)	64 (9.43)	30 (4.33)
	Sukkur	916	28.3	618 (67.54)	627 (68.33)	141 (15.45)	39 (4.27)	56 (6.10)	55 (5.85)	137 (15.00)	331 (36.17)	286 (31.19)	110 (12.03)	51 (5.61)
	**Provincial**	**6166**	**28.7**	**3414 (55.37)**	**3920 (63.57)**	**713 (11.56)**	**357 (5.79)**	**554 (8.98)**	**623 (10.10)**	**1442 (23.39)**	**1511 (24.50)**	**1213 (19.68)**	**1097 (17.79)**	**903 (14.64)**
KP	Bannu	620	28.97	588 (94.74)	459 (74.07)	64 (10.39)	22 (3.63)	24 (3.84)	50 (8.08)	137 (22.03)	136 (22.01)	194 (31.33)	93 (15.08)	59 (9.55)
	D.I.Khan	601	29.61	527 (87.72)	479 (79.80)	41 (6.90)	18 (3.02)	28 (4.61)	34 (5.67)	228 (39)	187 (31.19)	97 (16.23)	39 (6.52)	48 (8.07)
	Hazara	1198	28.75	1115 (93.14)	564 (47.13)	169 (14.11)	98 (8.17)	175 (14.62)	191 (15.97)	262 (21.87)	160 (13.33)	218 (18.22)	263 (21.98)	294 (24.60)
	Kohat	768	28.49	694 (90.37)	533 (69.37)	81 (10.50)	45 (5.85)	54 (7.11)	55 (7.18)	169 (21.95)	210 (27.28)	171 (22.31)	125 (16.32)	93 (12.14)
	Malakand	2591	28.03	2265 (87.42)	1717 (66.27)	336 (12.96)	196 (7.57)	179 (6.92)	163 (6.28)	655 (25.27)	567 (21.88)	448 (17.28)	561 (21.65)	361 (13.92)
	Mardan	1126	27.82	944 (83.86)	551 (48.95)	154 (13.64)	164 (14.55)	127 (11.28)	130 (11.58)	41 (3.65)	132 (11.71)	250 (22.22)	399 (35.42)	304 (27)
	Peshawar	2484	28.19	1799 (72.40)	1641 (66.05)	260 (10.48)	185 (7.44)	189 (7.60)	209 (8.43)	264 (10.65)	396 (15.94)	509 (20.51)	577 (23.21)	738 (29.7)
	**Provincial**	**9388**	**28.34**	**7932 (84.49)**	**5945 (63.33)**	**1105 (11.78)**	**728 (7.76)**	**776 (8.27)**	**833 (8.87)**	**1756 (18.70)**	**1788 (19.04)**	**1889 (20.12)**	**2058 (21.92)**	**1898 (20.22)**
Pooled	**National**	**31 210**	**28.56**	**21 745 (69.67)**	**16 230 (52.00)**	**4944 (15.84)**	**2748 (8.81)**	**3578 (11.46)**	**3709 (11.89)**	**6632 (21.25)**	**6409 (20.53)**	**6284 (20.13)**	**6235 (19.98)**	**5651 (18.11)**

*KP = Khyber–Phaktunkhwa*.

**Table 2.** Socio-Demographic Distribution (Weighted) of Women Without a Live Birth in the Past 2 Years (15-49 Years)

Socio-demographic distribution (women WITHOUT one or more live births in the previous 2 years)														
		Women (weighted)	Mean Age (years)	Rural dwellers n (%)	Education *n* (%)					Combined Wealth Index *n* (%)				
Province	Division				None/preschool	Primary	Middle	Secondary	Higher	Poorest	Second	Middle	Fourth	Richest
Punjab	Bhawalpur	2629	37.26	1876 (71.38)	1636 (62.22)	385 (14.64)	179 (6.82)	218 (8.29)	211 (8.03)	940 (35.75)	650 (24.72)	483 (18.39)	279 (10.63)	276 (10.51)
	DG Khan	2231	36.69	1832 (82.11)	1624 (72.82)	307 (13.76)	88 (3.95)	120 (5.40)	91 (4.08)	1211 (54.29)	551 (24.68)	254 (11.40)	142 (6.35)	73 (3.28)
	Faisalabad	3498	37.6	2079 (59.44)	1531 (43.77)	745 (21.30)	336 (9.62)	454 (12.98)	430 (12.31)	570 (16.31)	650 (18.57)	788 (22.52)	824 (23.55)	666 (19.05)
	Gujranwala	4174	37.85	2604 (62.37)	1327 (31.79)	963 (23.07)	580 (13.89)	771 (18.46)	534 (12.79)	138 (3.31)	508 (12.17)	967 (23.17)	1376 (32.96)	1185 (28.40)
	Lahore	4954	37.52	1338 (27.0)	1842 (37.19)	804 (16.23)	550 (11.10)	804 (16.24)	954 (19.25)	219 (4.42)	582 (11.76)	780 (15.74)	1314 (26.52)	2059 (41.56)
	Multan	3169	36.91	2192 (69.16)	1869 (58.98)	542 (17.11)	237 (7.47)	272 (8.57)	249 (7.86)	741 (23.38)	852 (26.87)	654 (30.65)	532 (16.79)	390 (12.30)
	Rawalpindi	2779	37.68	1694 (60.94)	880 (31.67)	612 (22.01)	293 (10.55)	514 (18.52)	479 (17.25)	133 (4.78)	324 (11.66)	551 (19.84)	739 (26.60)	1032 (37.12)
	Sahiwal	1775	37.6	1338 (75.42)	1011 (56.95)	371 (20.91)	142 (7.98)	130 (7.34)	121 (6.82)	394 (22.19)	557 (31.41)	380 (21.41)	274 (15.42)	170 (9.56)
	Sargodha	2238	37.48	1637 (73.18)	1294 (57.83)	432 (19.31)	158 (7.07)	199 (8.89)	154 (6.90)	549 (24.54)	631 (28.18)	536 (23.96)	352 (15.74)	170 (7.58)
	**Provincial**	**27447**	**37.44**	**16 590 (60.44)**	**13 015 (47.42)**	**5161 (18.80)**	**2563 (9.34)**	**3483 (12.69)**	**3224 (11.75)**	**4895 (17.83)**	**5304 (19.33)**	**5395 (19.66)**	**5832 (21.25)**	**6021 (21.94)**
Sindh	Hyderabad	2534	36.28	1504 (59.36)	1852 (73.08)	264 (10.41)	83 (3.27)	153 (6.03)	183 (7.21)	804 (31.74)	525 (20.73)	524 (20.67)	265 (10.48)	415 (16.38)
	Karachi	4552	35.54	292 (6.42)	1561 (34.29)	514 (11.30)	488 (10.73)	987 (21.69)	1001 (21.99)	24 (0.53)	42 (0.91)	524 (11.51)	1871 (41.10)	2091 (45.95)
	Larkana	1352	36.86	819 (60.58)	1043 (77.16)	145 (10.72)	28 (2.05)	62 (4.57)	74 (5.51)	284 (21.00)	478 (35.38)	358 (26.47)	165 (12.19)	67 (4.96)
	Mirpur Khas	990	35.76	764 (77.19)	807 (81.53)	64 (6.44)	24 (2.41)	38 (3.84)	57 (5.78)	521 (52.59)	201 (20.34)	157 (15.88)	59 (6.01)	51 (5.18)
	Shaheed Benazirabad	1184	36.7	834 (70.44)	891 (75.27)	159 (13.41)	39 (3.33)	50 (4.19)	45 (3.80)	261 (22.44)	365 (31.65)	353 (29.81)	129 (10.63)	65 (5.47)
	Sukkur	1261	36.56	827 (65.58)	896 (71.08)	193 (15.35)	33 (2.61)	66 (5.26)	72 (5.69)	170 (13.52)	414 (32.84)	418 (33.22)	171 (13.56)	86 (6.86)
	**Provincial**	**11 873**	**36.24**	**5040 (42.45)**	**7050 (59.38)**	**1339 (11.28)**	**695 (5.86)**	**1356 (11.42)**	**1432 (12.06)**	**2069 (17.43)**	**2035 (17.14)**	**2334 (19.66)**	**2658 (22.38)**	**2776 (23.38)**
KP	Bannu	933	35.99	878 (94.11)	781 (83.66)	55 (5.93)	24 (2.60)	30 (3.25)	43 (4.56)	220 (23.54)	285 (30.50)	245 (26.31)	109 (11.73)	74 (7.92)
	D.I.Khan	1176	36.2	993 (84.44)	1038 (88.21)	48 (4.08)	17 (1.47)	36 (3.05)	38 (3.20)	436 (37.07)	356 (30.27)	180 (15.34)	101 (8.56)	103 (8.76)
	Hazara	2674	36.34	2385 (89.17)	1647 (61.59)	360 (13.47)	150 (5.59)	262 (9.81)	255 (9.54)	697 (26.05)	412 (15.42)	404 (15.09)	494 (18.48)	667 (24.95)
	Kohat	1311	36.01	1116 (85.15)	1048 (79.97)	76 (5.79)	40 (3.04)	66 (5.05)	81 (6.15)	300 (22.85)	307 (23.39)	276 (21.07)	225 (17.18)	203 (15.51)
	Malakand	3609	35.15	3220 (89.22)	2833 (78.49)	333 (9.24)	128 (3.55)	154 (4.28)	161 (4.45)	920 (25.50)	763 (21.14)	697 (19.31)	699 (19.37)	529 (14.67)
	Mardan	1764	36.46	1428 (80.95)	1134 (64.30)	218 (12.36)	138 (7.84)	162 (9.16)	112 (6.34)	82 (4.64)	228 (12.90)	422 (23.91)	557 (31.59)	476 (26.96)
	Peshawar	3415	36.47	2351 (68.85)	2425 (71.01)	362 (10.61)	163 (4.79)	263 (6.91)	228 (6.67)	332 (9.73)	559 (16.38)	689 (20.18)	793 (23.23)	1041 (30.48)
	**Provincial**	**14 882**	**36.03**	**12 372 (83.13)**	**10 906 (73.28)**	**1453 (9.76)**	**661 (4.44)**	**947 (6.36)**	**917 (6.16)**	**2987 (20.07)**	**2909 (19.55)**	**2914 (19.58)**	**2980 (20.02)**	**3094 (20.79)**
Pooled	**National**	**54 202**	**36.79**	**34 002 (62.73)**	**30 971 (57.14)**	**7953 (14.67)**	**3919 (7.23)**	**5786 (10.67)**	**5573 (10.28)**	**9951 (18.36)**	**10 249 (18.91)**	**10 643 (19.64)**	**11 469 (21.16)**	**11 891 (21.94)**

*KP = Khyber-Phaktunkhwa*.

**Figure 1.**
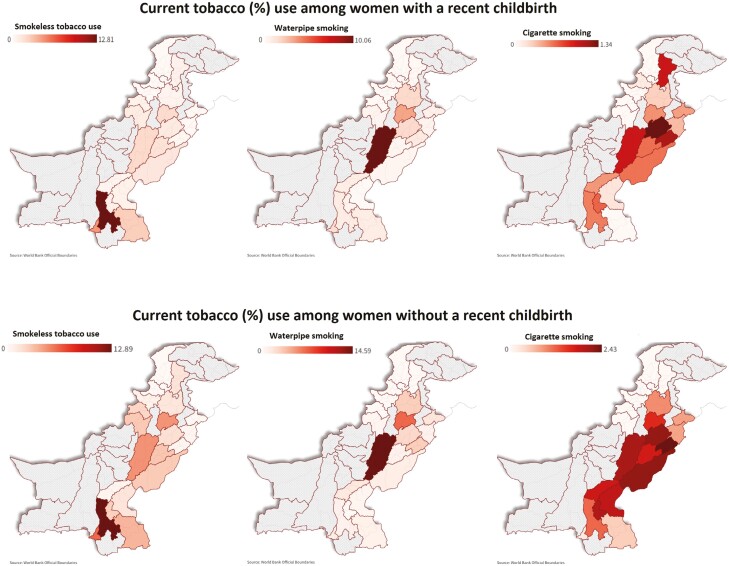
Prevalence estimates (weighted) of current tobacco use (smokeless tobacco use, cigarette smoking, and smoking waterpipe) among women with and without recent childbirth.

The pooled prevalence, across all administrative divisions, among women with recent childbirth was 1.4% (95% CI: 1.3–1.6) for current smokeless tobacco use, 0.4% (95% CI: 0.3–0.5) for current cigarette smoking, and 1.2% (95% CI: 1.05–1.4) for current waterpipe smoking. The pooled prevalence among women without recent childbirth was 2.5% (95% CI: 2.3–2.6), 1% (95% CI: 0.9–1.1), and 1.8% (95%CI: 1.6–1.9) for current smokeless tobacco use, cigarette smoking and waterpipe smoking, respectively. Across different administrative divisions of Pakistan, the prevalence of current smokeless tobacco use varied significantly (~13 percentage points) for both women with (0–12.8%) and without (0–12.9%) recent childbirth, while current cigarette smoking varied only a little, 0–1.3% among women with recent childbirth, and from 0% to 2.4% among women without recent childbirth. The prevalence of current waterpipe smoking also varied significantly among women with recent childbirth (0–10.1%) and among women without recent childbirth (0–14.6%). These variations in prevalence estimates are shown in Figure 1, and further reported with pooled provincial and national estimates in Tables S1 and S2.

**Figure 2.**
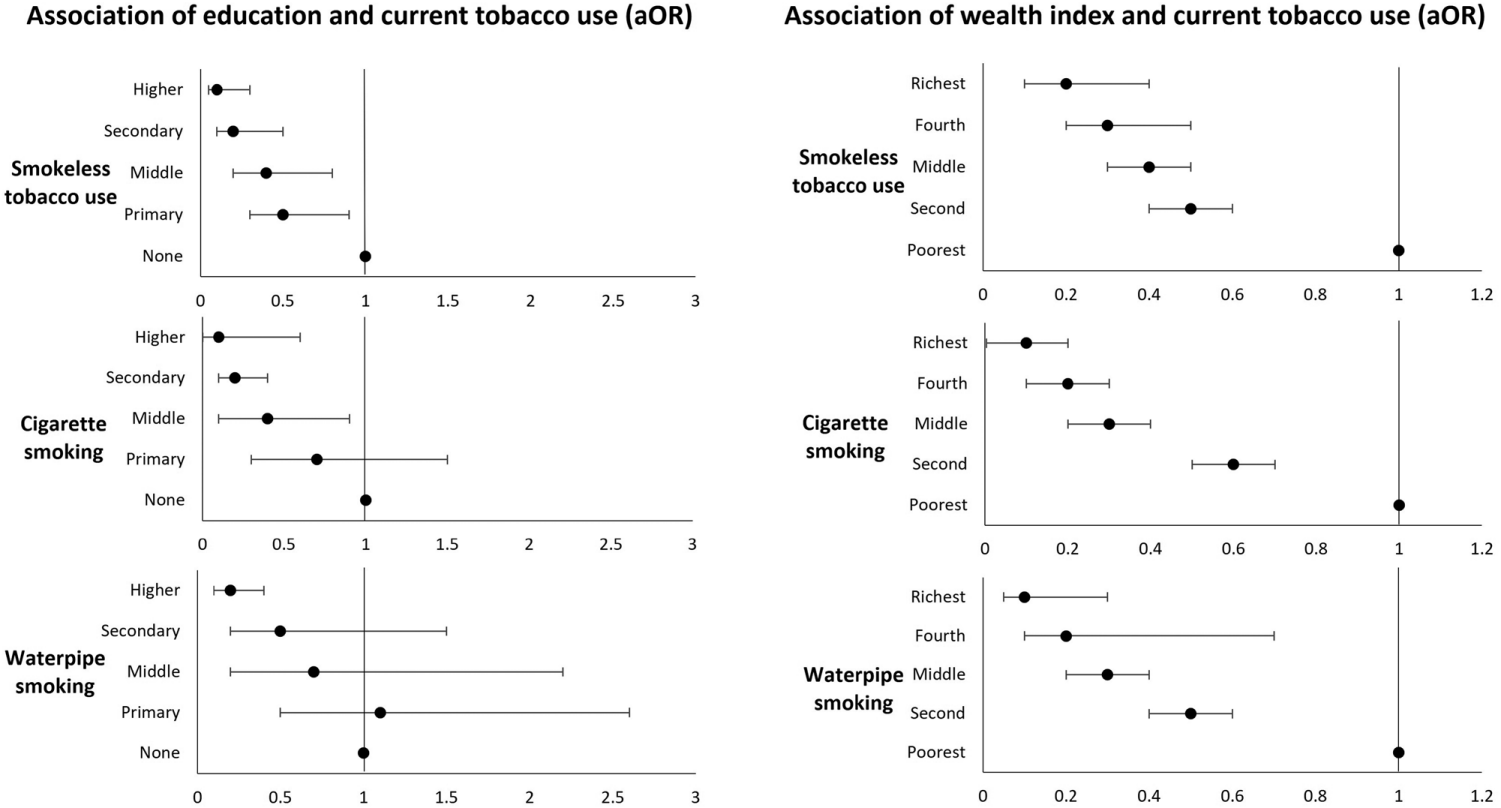
Forest plots showing associations between education and wealth index and current tobacco use by type. (cigarette smoking, smoking waterpipe and smokeless tobacco use).

Higher education was inversely associated with all forms of tobacco; odds were significantly lower for smokeless tobacco use (adjusted odd ratio (aOR) = 0.1, 95% CI: 0.05–0.3), cigarette smoking (aOR = 0.1, 95% CI: 0.00.6) and waterpipe smoking (aOR = 0.2, 95% CI: 0.1–0.4) as compared to non-users of that type of tobacco. The association between education and smokeless tobacco use was statistically significant across all levels; that is, with every incline in educational level, the odds of smokeless tobacco use declined significantly. Socio-economic status was also inversely associated with all three forms of tobacco use across each wealth quintile compared to the poorest quintile; odds for tobacco use decreased significantly with every increase in wealth quintile. This disparity gradient for education and socio-economic status is shown as forest plots in Figure 2. Further details of the regression analyses are listed in Table S3.

Birth weights were recorded for 15.4% (*n* = 4790/31 210) of the women with recent childbirth: 14% (*n* = 732) of these had the weight logged in the delivery record card, 70% (*n* = 3545) reported birth weight based on recall, and 11% (*n* = 513) did not provide any birth weight information. As most women did not have birth weight logged in the delivery record card, to make use of all available data, we used the birth weight reported both via delivery record and by recall. The mean birth weight was 3.55 kg (SD = 2.46). We found a statistically significant difference in birth weight of about 0.83 kg (95% CI: −1.6 to 0.1) in children born to mothers who, at the time of the interview, smoked waterpipe compared to non-tobacco users. While for smokeless tobacco use (0.33 kg, 95% CI: −0.9 to 0.3) and cigarette smoking (0.35 kg, 95% CI: −1.5 to 2.2) the differences were statistically not significant (Table 3).

**Table 3.** Estimates of Multiple Linear Regression Analysis for Birth Weight and Current Smokeless Tobacco Use, Cigarette Smoking, and Waterpipe Smoking

Multiple linear regression analysis		
	Coef. (95% CI)	*p*-Value
**Current tobacco use (reference = non-tobacco users)**		
**Exclusive smokeless tobacco use**	−0.33 (−0.9, 0.3)	.29
**Exclusive cigarette smoking**	0.35 (−1.5, 2.2)	.7
**Exclusive waterpipe smoking**	−0.83 (−1.6, −0.1)	.04
**Birth weight constant**	3.56 (3.48–3.63)	.00

*Note: Total observations = 4507; the absolute numbers of women who exclusively used* smokeless tobacco *were 63, smoked cigarettes were 7, and smoked waterpipe were 37.*

## Discussion

We utilized data from a large representative sample of women and estimated the prevalence for three types of tobacco use (smokeless tobacco, cigarettes, and waterpipe) according to the history of recent childbirth. The data suggests that there is variation in prevalence between provinces and by type of tobacco, with cigarette smoking as least prevalent across all three provinces, smokeless tobacco use most prevalent in Sindh (~6%) and waterpipe smoking most prevalent in Punjab (~3%). Furthermore, the prevalence of tobacco use varied even more significantly across the administrative divisions; 13 percentage points for smokeless tobacco use in Sindh and 15 percentage points for waterpipe smoking in Punjab. Among a subset of women (31 210) with recent childbirth, similar prevalence patterns were observed. The findings are worrying because it indicates that there is little change in tobacco use behavior around pregnancy and early motherhood. Furthermore, the high prevalence of tobacco use in certain geographical areas (administrative divisions) may go unnoticed due to relatively lower national estimates and the opportunity to intervene and benefit public health may not be available. Potential factors influencing these variations include cultural association and social acceptance of specific tobacco use among women in certain regions and limited awareness of tobacco-related harms.^13,34^ High smokeless tobacco use in certain areas may also be driven by easy access to cheap products manufactured locally. Additionally, there may be variations in regulating tobacco products and support for women to reduce or quit tobacco.^13,20^ This highlights the need for tailored public health measures to increase awareness and offer support to women in reducing or quitting tobacco, across all types of tobacco products.

Higher education was significantly associated with a decrease in tobacco use. This inverse association was most distinct and significant across all educational levels with smokeless tobacco use; aOR dropped from 0.5 to 0.1 from primary to higher level education compared to those with no formal education. Socioeconomic status was also significantly associated with a reduction in all forms of tobacco use. These education and socioeconomic disparities are consistent with existing literature.^11,35^ However, the significantly lower odds of tobacco use found among women with high levels of education in Pakistan is an important finding for a country where female literacy remains less than 50%.^36^ This is crucial because tobacco use and social inequalities are recognized as factors that exacerbate health disparities.^37,38^ These disparities occur due to easy access to cheap unregulated and highly toxic tobacco products in poor neighborhoods, limited awareness about tobacco-related harms, poor health literacy, and access to public health initiatives among those with poor educational attainment, which can in turn lead to lower rates of tobacco cessation, thus further widening of the health gap.^13,37^ To assist women in reducing or quitting tobacco use, public health measures should be designed to incorporate socio-cultural contexts and consider implementing pictorial warnings not only on cigarette packaging but also across all forms of tobacco products.

We found a statistically significant difference (830 g) in birth weight for babies born to mothers who at the time of the interview smoked waterpipe compared to children born to mothers who did not use any form of tobacco. There is some literature on this suggesting the association of waterpipe smoking with low birth weight. A study conducted among pregnant women in Egypt reported lower mean birth weight (*p*-value <.001) compared to non-smokers, however women who smoked included cigarette and waterpipe smoking, though waterpipe was more prevalent.^39^ Another study based on a prospective cohort in Iran, reported the adjusted risk of low birth weight to be twice among waterpipe smokers.^40^ The possible explanation could be the exposure to smoke that contains toxicants from the tobacco product itself in addition to the burning of charcoal which is required when using a waterpipe.^41^ Waterpipe smoking also results in high exposures to carbon monoxide and polycyclic aromatic hydrocarbons, which are known carcinogens and teratogens.^41,42^ In terms of smokeless tobacco use and birth weight, our findings suggest that the birth weight was about 330 g less for children born to mothers who at the time of the interview used smokeless tobacco compared to children born to mothers who did not use any form of tobacco, however this was not statistically significant. One other study conducted in Pakistan also reported no statistically significant association between smokeless tobacco use before conception and birth weight (RR = 0.96, 95% CI 0.7–1.3).^43^ However, other studies have reported a weak to moderate evidence on smokeless tobacco use during pregnancy and low birth weight.^44,45^ Gupta et al. estimated smokeless tobacco use and birth weight based on a prospective cohort of 1217 women and reported a reduction of 105 g (*p* value = .006) in birth weight, which was consistent after adjustment for gestational age.^45^ The strong evidence on the effect of combustible tobacco on birth weight, may not be applicable for smokeless tobacco, and needs further longitudinal studies, accounting for the diverse smokeless tobacco products and amount of consumption. Furthermore, our findings of birth weight and cigarette smoking were not statistically significant, possibly due to very small number of smokers in our cohort, however this association is already well-established in the literature.^2,46^

We highlight a significant missed opportunity; most newborns were either not weighed at birth or did not have a record of it. This is consistent with previous MICS datasets and warrants the urgent need for better recording of birth events to help improve maternal and child health.^47^ Especially with diverse tobacco use in South Asia and the dearth of literature, such large, nationally representative data hold significant value in understanding the effect of these forms of tobacco on peri-natal outcomes.

### Strengths

The prevalence estimates of tobacco use (smokeless tobacco, cigarette smoking and waterpipe smoking) from a nationally representative sample along with estimates based on administrative divisions is novel. Certain areas with high levels of tobacco use (geographical location and type of tobacco) need further exploration to understand the contextual factors. The association of smokeless tobacco use and waterpipe smoking with birth weight from a representative sample is also a novel finding.

### Limitations

First, among women with recent childbirth, <15% had birth weight of their child recorded which indicates the challenges in obtaining this data. However, we compared the socio-demographic characteristics of women who reported birth weight based on delivery record and recall (Table S4) and found that women who provided birth weight data based on recall were residing more in rural areas, were less educated and belonged to low socio-economic status. Second, this is a cross-sectional survey and hence causal links cannot be assumed. In addition, for the analysis looking at the association between birth weight and tobacco use, there is a major assumption that those who are currently using tobacco might have also used the same form of tobacco during their pregnancy. Furthermore, we did not adjust for several factors that affect birth weight (eg, gestational age, maternal weight) due to the unavailability of such data. Further research such as a large cohort is required to understand the effect of different forms of tobacco on birth weight. Only a few women reported cigarette smoking, which meant that we did not have sufficient power to provide precise estimates of this sub-group.

## Conclusions

A high prevalence of smokeless tobacco use (13%) and waterpipe smoking (15%) in certain parts of Pakistan emphasizes the need for tailored and targeted tobacco control interventions. Further contextual research is warranted to understand the reasons for such high tobacco use in these areas of Pakistan and what measures could help reduce its use. The statistically significant association between waterpipe smoking and low birth weight is important for policy given the gaining popularity of waterpipe smoking among youth including women. Further research is warranted to understand the impact of smokeless tobacco use on birth outcomes through longitudinal studies accounting for diverse products, its constituents and amount of consumption.

## Supplementary material

Supplementary material is available at *Nicotine and Tobacco Research* online.

ntae016_suppl_Supplementary_Tables_S1-S4

## Data Availability

The data that support the findings of this study are available from the Multiple Indicator Cluster Surveys (https://mics.unicef.org/) upon request.

## References

[1] Marufu TC , Ahankari A, Coleman T, Lewis S. Maternal smoking and the risk of still birth: systematic review and meta-analysis. BMC Public Health. 2015;15(1):1–15. 25885887 10.1186/s12889-015-1552-5 PMC4372174

[2] Veisani Y , Jenabi E, Delpisheh A, Khazaei S. Effect of prenatal smoking cessation interventions on birth weight: meta-analysis. J Matern Fetal Neonatal Med. 2019;32(2):332–338. 28889768 10.1080/14767058.2017.1378335

[3] Tolosa JE , Scherman A, Stamilio DM, McEvoy CT. Tobacco and nicotine exposure prevention in pregnancy: a priority to improve perinatal and maternal outcomes. Am J Obstet Gynecol MFM. 2019;1(1):19–23. 33319752 10.1016/j.ajogmf.2019.03.005 PMC8023387

[4] Wong MK , Barra NG, Alfaidy N, Hardy DB, Holloway AC. Adverse effects of perinatal nicotine exposure on reproductive outcomes. Reproduction. 2015;150(6):R185–R193. 26432348 10.1530/REP-15-0295

[5] Kondo T , Nakano Y, Adachi S, Murohara T. Effects of tobacco smoking on cardiovascular disease. Circ J. 2019;83(10):1980–1985. 31462607 10.1253/circj.CJ-19-0323

[6] CDC Vitalsigns. Cancer and tobacco use-Tobacco use causes many cancers 2016. https://www.cdc.gov/vitalsigns/pdf/2016-11-vitalsigns.pdf. Accessed January 15, 2023.

[7] Asma S. The GATS atlas: Global Adult Tobacco Survey. 2015. https://stacks.cdc.gov/view/cdc/51993. Accessed October 19, 2022. 10.3390/ijerph121215004 PMC4690940 26670238

[8] Parascandola M , Bloch M. The global laboratory of tobacco control: research to advance tobacco cessation in LMICs. J Smok Cessat. 2016;11(2):70–77.

[9] Maziak W , Taleb ZB, Bahelah R, et al. The global epidemiology of waterpipe smoking. Tob Control. 2015;24(Suppl 1):i3–i12. 25298368 10.1136/tobaccocontrol-2014-051903 PMC4345835

[10] Babaie J , Ahmadi A, Abdollahi G, Doshmangir L. Preventing and controlling water pipe smoking: a systematic review of management interventions. BMC Public Health 2021;21(1):1–12. 33632181 10.1186/s12889-021-10306-w PMC7908788

[11] Sreeramareddy CT , Pradhan PMS, Mir IA, Sin S. Smoking and smokeless tobacco use in nine South and Southeast Asian countries: prevalence estimates and social determinants from Demographic and Health Surveys. Popul Health Metr. 2014;12(1):1–16. 25183954 10.1186/s12963-014-0022-0 PMC4151025

[12] Shukla R , Kanaan M, Siddiqi K. Tobacco use among 1 310 716 women of reproductive age (15–49 Years) in 42 low-and middle-income countries: secondary data analysis from the 2010–2016 demographic and health surveys. Nicotine Tob Res. 2021;23(12):2019–2027. 34291296 10.1093/ntr/ntab131 PMC8849114

[13] Khan MT , Hashmi S, Zaheer S, et al. Burden of waterpipe smoking and chewing tobacco use among women of reproductive age group using data from the 2012–13 Pakistan demographic and health survey. BMC Public Health. 2015;15(1):1–8. 26563874 10.1186/s12889-015-2433-7 PMC4643522

[14] Kendrick PJ , Reitsma MB, Abbasi-Kangevari M, et al. Spatial, temporal, and demographic patterns in prevalence of chewing tobacco use in 204 countries and territories, 1990–2019: a systematic analysis from the Global Burden of Disease Study 2019. Lancet Public Health. 2021;6(7):e482–ee99. 34051920 10.1016/S2468-2667(21)00065-7 PMC8251505

[15] Flora MS , Mascie-Taylor C, Rahman M. Gender and locality differences in tobacco prevalence among adult Bangladeshis. Tob Control. 2009;18(6):445–450. 19679888 10.1136/tc.2008.028142 PMC2778071

[16] Dadipoor S , Kok G, Aghamolaei T, et al. Factors associated with hookah smoking among women: A systematic review. Tob Prev Cessat. 2019;5(August):26. 32411889 10.18332/tpc/110586 PMC7205165

[17] Kakde S , Bhopal R, Jones C. A systematic review on the social context of smokeless tobacco use in the South Asian population: implications for public health. Public Health. 2012;126(8):635–645. 22809493 10.1016/j.puhe.2012.05.002

[18] Singh PK , Singh L, Wehrmeister FC, et al. Prevalence of smoking and smokeless tobacco use during breastfeeding: a cross-sectional secondary data analysis based on 0.32 million sample women in 78 low-income and middle-income countries. EClinicalMedicine. 2022;53(November):101660. 36159043 10.1016/j.eclinm.2022.101660 PMC9489519

[19] GATS Pakistan Report. Pakistan Health Research Council;2014.

[20] Iqbal N , Irfan M, Ashraf N, Awan S, Khan JA. Prevalence of tobacco use among women: a cross sectional survey from a squatter settlement of Karachi, Pakistan. BMC Res Notes. 2015;8(1):1–5. 26400484 10.1186/s13104-015-1455-7 PMC4581439

[21] Nisar N , Billoo N, Gadit AA, et al. Pattern of tobacco consumption among adult women of low socioeconomic community Karachi, Pakistan. J Pak Med Assoc. 2005;55(3):111–114. 15852747

[22] Sinha DN , Suliankatchi RA, Gupta PC, et al. Global burden of all-cause and cause-specific mortality due to smokeless tobacco use: systematic review and meta-analysis. Tob Control. 2018;27(1):35–42. 27903956 10.1136/tobaccocontrol-2016-053302

[23] Siddiqi K , Husain S, Vidyasagaran A, et al. Global burden of disease due to smokeless tobacco consumption in adults: an updated analysis of data from 127 countries. BMC Med. 2020;18(1):1–22. 32782007 10.1186/s12916-020-01677-9 PMC7422596

[24] Asthana S , Labani S, Kailash U, Sinha DN, Mehrotra R. Association of smokeless tobacco use and oral cancer: a systematic global review and meta-analysis. Nicotine Tob Res. 2019;21(9):1162–1171. 29790998 10.1093/ntr/nty074

[25] Vidyasagaran AL , Siddiqi K, Kanaan M. Use of smokeless tobacco and risk of cardiovascular disease: a systematic review and meta-analysis. Eur J Prev Cardiol. 2016;23(18):1970–1981. 27256827 10.1177/2047487316654026

[26] Mamtani R , Cheema S, Sheikh J, et al. Cancer risk in waterpipe smokers: a meta-analysis. Int J Public Health. 2017;62(1):73–83. 27421466 10.1007/s00038-016-0856-2 PMC5288449

[27] Raad D , Gaddam S, Schunemann HJ, et al. Effects of water-pipe smoking on lung function: a systematic review and meta-analysis. Chest. 2011;139(4):764–774. 20671057 10.1378/chest.10-0991

[28] Patel MP , Khangoora VS, Marik PE. A review of the pulmonary and health impacts of hookah use. Ann Am Thorac Soc 2019;16(10):1215–1219. 31091965 10.1513/AnnalsATS.201902-129CME

[29] Qasim H , Alarabi AB, Alzoubi KH, et al. The effects of hookah/waterpipe smoking on general health and the cardiovascular system. Environ Health Prev Med. 2019;24(1):1–17. 31521105 10.1186/s12199-019-0811-y PMC6745078

[30] England LJ , Kim SY, Tomar SL, et al. Non-cigarette tobacco use among women and adverse pregnancy outcomes. Acta Obstet Gynecol Scand. 2010;89(4):454–464. 20225987 10.3109/00016341003605719 PMC5881107

[31] Bureau of Statistics Punjab PDB, Government of the Punjab. Multiple Indicator Cluster Survey Punjab, 2017-18, Survey Findings Report. UNICEF, 2018.

[32] Rustein SO , Johnson K. The DHS wealth index. 2004.

[33] Filmer D , Pritchett LH. Estimating wealth effects without expenditure data--or tears: an application to educational enrollments in states of India. Demography. 2001;38(1):115–132. 11227840 10.1353/dem.2001.0003

[34] Zubair F , Husnain MI, Zhao T, Ahmad H, Khanam R. A gender-specific assessment of tobacco use risk factors: evidence from the latest Pakistan demographic and health survey. BMC Public Health. 2022;22(1):1133. 35668426 10.1186/s12889-022-13574-2 PMC9172179

[35] Sreeramareddy CT , Harper S, Ernstsen L. Educational and wealth inequalities in tobacco use among men and women in 54 low-income and middle-income countries. Tob Control. 2018;27(1):26–34. 27885168 10.1136/tobaccocontrol-2016-053266

[36] Tagar HK , Abro LT, Chandani AM, Khoso ZA, Sohoo MN. The low female literacy trends: a critical challenge of human development in Pakistan (Major Obstacles and Way Forward). Arch Bus Res. 2019;7(6):88–97.

[37] Mentis A-FA. Social determinants of tobacco use: towards an equity lens approach. Tob Prev Cessat. 2017;3(March):7. 32432182 10.18332/tpc/68836 PMC7232809

[38] Bandyopadhyay A , Irfan M. Educational and wealth inequalities in smokeless tobacco use: an analysis of rural-urban areas of Bangladesh and India. Subst Abuse. 2019;13(January):1178221818825074. 30906193 10.1177/1178221818825074 PMC6421618

[39] El-Shahawy O , Labib K, Stevens E, et al. Exclusive and dual cigarette and hookah smoking is associated with adverse perinatal outcomes among pregnant women in Cairo, Egypt. Int J Environ Res Public Health. 2021;18(24):12974. 34948585 10.3390/ijerph182412974 PMC8701206

[40] Nematollahi S , Mansournia MA, Foroushani AR, et al. The effects of water-pipe smoking on birth weight: a population-based prospective cohort study in southern Iran. Epidemiol Health. 2018;40(March):e2018008. 29529859 10.4178/epih.e2018008 PMC5968205

[41] WHO Study Group on Tobacco Product Regulation. Advisory note: waterpipe tobacco smoking: health effects, research needs and recommended actions by regulators. World Health Organization, 2015.

[42] Patel AB , Shaikh S, Jain KR, Desai C, Madamwar D. Polycyclic aromatic hydrocarbons: sources, toxicity, and remediation approaches. Front Microbiol. 2020;11:562813. 33224110 10.3389/fmicb.2020.562813 PMC7674206

[43] Aziz Ali S , Khan U, Abrejo F, et al. Use of smokeless tobacco before conception and its relationship with maternal and fetal outcomes of pregnancy in Thatta, Pakistan: Findings from women first study. Nicotine Tob Res. 2021;23(8):1291–1299. 33084903 10.1093/ntr/ntaa215 PMC8360631

[44] Inamdar AS , Croucher RE, Chokhandre MK, Mashyakhy MH, Marinho VC. Maternal smokeless tobacco use in pregnancy and adverse health outcomes in newborns: a systematic review. Nicotine Tob Res. 2014;17(9):1058–1066. 25534929 10.1093/ntr/ntu255

[45] Gupta PC , Sreevidya S. Smokeless tobacco use, birth weight, and gestational age: population based, prospective cohort study of 1217 women in Mumbai, India. BMJ. 2004;328(7455):1538. 15198947 10.1136/bmj.38113.687882.EB PMC437147

[46] Di H-K , Gan Y, Lu K, et al. Maternal smoking status during pregnancy and low birth weight in offspring: systematic review and meta-analysis of 55 cohort studies published from 1986 to 2020. World J Pediatr. 2022;18(3):176–185. 35089538 10.1007/s12519-021-00501-5

[47] Biks GA , Blencowe H, Hardy VP, et al. Birthweight data completeness and quality in population-based surveys: EN-INDEPTH study. Popul Health Metr. 2021;19(1):1–16. 33557859 10.1186/s12963-020-00229-w PMC7869202

