# Supplementary material for: The Diversity in Tobacco Use Among Women of Reproductive Age (15–49 Years) in Pakistan: A Secondary Analysis of a Multiple Indicator Cluster Survey 2016–2018
Source: Nicotine Tob Res. 2024 Feb 1;26(7):931–9. doi: 10.1093/ntr/ntae016 (PMC11190045; doi:10.1093/ntr/ntae016)
Supplement: ntae016_suppl_Supplementary_Tables_S1-S4 [file ntae016_suppl_supplementary_tables_s1-s4.docx]

**The diversity in tobacco use among women of reproductive age (15-49 years) in Pakistan: A secondary analysis of a Multiple Indicator Survey (MICS)**

___________________________________________________________________________________________________________________________________

**Supplementary**

**Supplemental Data:**

**Table S1:** Prevalence estimates (weighted) and 95% confidence intervals of tobacco use among women with a live birth in the past 2 years, stratified by provinces and divisions.

**Table S2:** Prevalence estimates (weighted) and 95% confidence intervals of tobacco use among women without a live birth in the past 2 years, stratified by provinces and divisions.

**Table S3**: Estimates of logistic regression analyses for current tobacco use (as an outcome) and socio-demographic characteristics (education, area of residence, wealth index and age) (as explanatory variables) among women of reproductive age (15-49 years). Current tobacco use was investigated for each type (ST use, cigarette smoking, and waterpipe smoking) separately, reference group in each case was those that are not currently using that type of tobacco.

**Table S4**: Sociodemographic distribution (weighted) of women with a live birth in the past 2 years based on the birth weight data.

**Table S1:** Prevalence estimates (weighted) and 95% confidence intervals of tobacco use among women with a live birth in the past 2 years, stratified by provinces and divisions.

| **Tobacco use among women WITH a live birth in the previous 2 years** | | | | | | | | | | | | | | | | | | | |
| --- | --- | --- | --- | --- | --- | --- | --- | --- | --- | --- | --- | --- | --- | --- | --- | --- | --- | --- | --- |
|  |  | **Ever ST use** | | | **Current ST use** | | | **Ever cigarette smoking** | | | **Current cigarette smoking** | | | **Ever waterpipe smoking** | | | **Current waterpipe smoking** | | |
| **Province** | **Division** | **n** | **%** | **95% CI** | **n** | **%** | **95% CI** | **n** | **%** | **95% CI** | **n** | **%** | **95% CI** | **n** | **%** | **95% CI** | **n** | **%** | **95% CI** |
| **Punjab** | Bhawalpur | 27 | 1.74 | 1.15 - 2.62 | 21 | 1.38 | 0.86 - 2.19 | 18 | 1.18 | 0.74 - 1.87 | 10 | 0.61 | 0.32 - 1.15 | 11 | 0.7 | 0.38 - 1.28 | 4 | 0.27 | 0.1 - 0.73 |
|  | DG Khan | 45 | 2.57 | 1.94 - 3.41 | 37 | 2.11 | 1.52 - 2.92 | 51 | 2.9 | 2.12 - 3.95 | 17 | 1 | 0.6 - 1.66 | 198 | 11.36 | 9.68 - 13.27 | 175 | 10.06 | 8.53 - 11.84 |
|  | Faisalabad | 31 | 1.61 | 1.11 - 2.33 | 22 | 1.17 | 0.74 - 1.83 | 57 | 3 | 2.31 - 3.89 | 25 | 1.34 | 0.9 - 1.97 | 47 | 2.45 | 1.79 - 3.34 | 31 | 1.63 | 1.1 - 2.42 |
|  | Gujranwala | 17 | 0.79 | 0.48 - 1.28 | 4 | 0.18 | 0.005 - 0.6 | 38 | 1.7 | 1.25 - 2.3 | 10 | 0.46 | 0.29 - 0.75 | 30 | 1.35 | 0.97 - 1.88 | 13 | 0.59 | 0.36 - 0.96 |
|  | Lahore | 20 | 0.78 | 0.46 - 1.31 | 4 | 0.15 | 0.005 - 0.43 | 44 | 1.68 | 1.19 - 2.38 | 9 | 0.35 | 0.18 - 0.68 | 31 | 1.18 | 0.8 - 1.72 | 12 | 0.44 | 0.25 - 0.78 |
|  | Multan | 31 | 1.65 | 1.15 - 2.37 | 21 | 1.13 | 0.71 - 1.8 | 34 | 1.81 | 1.28 - 2.55 | 11 | 0.62 | 0.34 - 1.13 | 11 | 0.62 | 0.35 - 1.09 | 5 | 0.27 | 0.11 - 0.63 |
|  | Rawalpindi | 18 | 1.36 | 0.87 - 2.1 | 7 | 0.5 | 0.25 - 1.01 | 23 | 1.7 | 1.17 - 2.47 | 4 | 0.27 | 0.1 - 0.7 | 33 | 2.45 | 1.78 - 3.36 | 22 | 1.64 | 1.12 - 2.39 |
|  | Sahiwal | 8 | 0.71 | 0.33 - 1.52 | 3 | 0.26 | 0.008 - 0.8 | 22 | 1.99 | 1.27 - 3.11 | 12 | 1.13 | 0.62 - 2.04 | 15 | 1.37 | 0.82 - 2.29 | 10 | 0.92 | 0.48 - 1.77 |
|  | Sargodha | 32 | 2.46 | 1.73 - 3.49 | 26 | 2.03 | 1.36 - 3.01 | 20 | 1.56 | 0.97 - 2.5 | 7 | 0.51 | 0.24 - 1.11 | 59 | 4.53 | 3.52 - 5.82 | 43 | 3.31 | 2.49 - 4.38 |
|  | **Provincial** | **229** | **1.46** | **1.28 - 1.67** | **145** | **0.93** | **0.78 - 1.1** | **307** | **1.96** | **1.74 - 2.2** | **105** | **0.67** | **0.56 - 0.82** | **435** | **2.78** | **2.51 - 3.08** | **315** | **2.01** | **1.79 - 2.27** |
| **Sindh** | Hyderabad | 249 | 17.91 | 15.77 - 20.27 | 178 | 12.81 | 11.02 - 14.84 | 13 | 0.91 | 0.56 - 1.49 | 8 | 0.57 | 0.29 - 1.13 | 25 | 1.82 | 1.2 - 2.75 | 11 | 0.81 | 0.4 - 1.61 |
|  | Karachi | 115 | 7.54 | 5.97 - 9.48 | 72 | 4.71 | 3.37 - 6.55 | 17 | 1.09 | 0.63 - 1.88 | 1 | 0.006 | 0.009 - 0.47 | 47 | 3.05 | 2.14 - 4.34 | 22 | 1.43 | 0.8 - 2.53 |
|  | Larkana | 15 | 1.48 | 0.95 - 2.3 | 5 | 0.49 | 0.23 - 1.02 | 14 | 1.4 | 0.77 - 2.51 | 5 | 0.49 | 0.2 - 1.22 | 21 | 2.14 | 1.45 - 3.17 | 7 | 0.7 | 0.34 - 1.45 |
|  | Mirpur Khas | 27 | 4.17 | 2.71 - 6.35 | 17 | 2.65 | 1.53 - 4.55 | 2 | 0.34 | 0.004 - 2.42 | 0 | 0 | 0 | 11 | 1.65 | 0.62 - 4.3 | 5 | 0.79 | 0.23 - 2.75 |
|  | Shaheed Benazirabad | 12 | 1.78 | 0.87 - 3.6 | 5 | 0.8 | 0.23 - 2.69 | 9 | 1.36 | 0.72 - 2.57 | 5 | 0.66 | 0.26 - 1.7 | 8 | 1.11 | 0.49 - 2.47 | 3 | 0.42 | 0.009 - 1.81 |
|  | Sukkur | 8 | 0.91 | 0.43 - 1.93 | 6 | 0.66 | 0.27 - 1.59 | 17 | 1.88 | 0.72 - 4.83 | 1 | 0.16 | 0.002 - 1.12 | 0 | 0 | 0 | 0 | 0 | 0 |
|  | **Provincial** | **426** | **6.91** | **6.2 - 7.7** | **283** | **4.59** | **4.0 - 5.28** | **72** | **1.17** | **0.85 - 1.6** | **20** | **0.32** | **0.21 - 0.5** | **112** | **1.81** | **1.46 - 2.25** | **48** | **0.78** | **0.54 - 1.12** |
| **KP** | Bannu | 7 | 1.13 | 0.45 - 2.8 | 4 | 0.61 | 0.23 - 1.62 | 0 | 0 | 0 | 0 | 0 | 0 | 1 | 0.19 | 0.002 - 1.39 | 0 | 0 | 0 |
|  | D.I.Khan | 8 | 1.37 | 0.69 - 2.69 | 6 | 1.05 | 0.46 - 2.39 | 0 | 0 | 0 | 0 | 0 | 0 | 4 | 0.71 | 0.18 - 2.74 | 2 | 0.37 | 0.005 - 2.61 |
|  | Hazara | 20 | 1.68 | 0.98 - 2.86 | 7 | 0.6 | 0.26 - 1.37 | 17 | 1.45 | 0.89 - 2.36 | 1 | 0.004 | 0.001 - 0.34 | 8 | 0.7 | 0.34 - 1.41 | 2 | 0.14 | 0.003 - 0.58 |
|  | Kohat | 3 | 0.42 | 0.17 - 1.03 | 0 | 0 | 0 | 10 | 1.29 | 0.65 - 2.54 | 0 | 0 | 0 | 6 | 0.78 | 0.36 - 1.64 | 1 | 0.007 | 0.001 - 0.56 |
|  | Malakand | 6 | 0.21 | 0.001 - 0.43 | 1 | 0.003 | 0.001 - 0.12 | 17 | 0.67 | 0.43 - 1.04 | 1 | 0.003 | 0.001 - 0.12 | 13 | 0.48 | 0.22 - 1.07 | 0 | 0 | 0 |
|  | Mardan | 5 | 0.42 | 0.14 - 1.29 | 0 | 0 | 0 | 8 | 0.66 | 0.28 - 1.56 | 0 | 0 | 0 | 6 | 0.52 | 0.19 - 1.38 | 0 | 0 | 0 |
|  | Peshawar | 7 | 0.29 | 0.11 - 0.79 | 1 | 0.003 | 0.001 - 0.25 | 13 | 0.53 | 0.23 - 1.18 | 3 | 0.11 | 0.001 - 0.79 | 15 | 0.59 | 0.28 - 1.23 | 3 | 0.11 | 0.001 - 0.8 |
|  | **Provincial** | **56** | **0.6** | **0.44 - 0.81** | **19** | **0.2** | **0.13 - 0.33** | **65** | **0.7** | **0.53 - 0.92** | **5** | **0.04** | **0.001 - 0.08** | **53** | **0.56** | **0.4 - 0.8** | **8** | **0.08** | **0.002 - 0.22** |
| **Pooled** | **National** | **711** | **2.28** | **2.06 - 2.52** | **447** | **1.43** | **1.26 - 1.63** | **444** | **1.42** | **1.28 - 1.58** | **130** | **0.41** | **0.35 - 0.5** | **600** | **1.92** | **1.74 - 2.12** | **371** | **1.19** | **1.05 - 1.35** |

*KP = Khyber-Phaktunkhwa, ST = smokeless tobacco*

**Table S2:** Prevalence estimates (weighted) and 95% confidence intervals of tobacco use among women without a live birth in the past 2 years, stratified by provinces and divisions.

| **Tobacco use among women WITHOUT a live birth in the previous 2 years** | | | | | | | | | | | | | | | | | | | |
| --- | --- | --- | --- | --- | --- | --- | --- | --- | --- | --- | --- | --- | --- | --- | --- | --- | --- | --- | --- |
|  |  | **Ever ST use** | | | **Current ST use** | | | **Ever cigarette smoking** | | | **Current cigarette smoking** | | | **Ever waterpipe smoking** | | | **Current waterpipe smoking** | | |
| **Province** | **Division** | **n** | **%** | **95% CI** | **n** | **%** | **95% CI** | **n** | **%** | **95% CI** | **n** | **%** | **95% CI** | **n** | **%** | **95% CI** | **n** | **%** | **95% CI** |
| **Punjab** | Bhawalpur | 83 | 3.14 | 2.41 - 4.08 | 73 | 2.8 | 2.12 - 3.71 | 82 | 3.13 | 2.36 - 4.14 | 58 | 2.23 | 1.62 - 3.05 | 44 | 1.69 | 1.19 - 2.4 | 26 | 1 | 0.65 - 1.52 |
|  | DG Khan | 116 | 5.18 | 4.1 - 6.52 | 108 | 4.84 | 3.8 - 6.15 | 104 | 4.66 | 3.79 - 5.7 | 46 | 2.08 | 1.53 0 2.83 | 371 | 16.62 | 14.62 - 18.83 | 325 | 14.59 | 12.74 - 16.65 |
|  | Faisalabad | 83 | 2.38 | 1.9 - 2.96 | 63 | 1.82 | 1.4 - 2.34 | 142 | 4.05 | 3.37 - 4.87 | 76 | 2.21 | 1.73 - 2.82 | 133 | 3.79 | 3.15 - 4.55 | 87 | 2.48 | 1.97 - 3.11 |
|  | Gujranwala | 27 | 0.64 | 0.43 - 0.96 | 7 | 0.18 | 0.008 - 0.39 | 74 | 1.78 | 1.43 - 2.21 | 34 | 0.82 | 0.59 - 1.13 | 121 | 2.91 | 2.49 - 3.39 | 69 | 1.65 | 1.36 - 2.01 |
|  | Lahore | 38 | 0.78 | 0.52 - 1.15 | 16 | 0.32 | 0.18 - 0.56 | 149 | 3.01 | 2.45 - 3.69 | 39 | 0.8 | 0.55 - 1.16 | 107 | 2.16 | 1.74 - 2.68 | 36 | 0.73 | 0.52 - 1.03 |
|  | Multan | 129 | 4.07 | 3.39 - 4.88 | 96 | 3.03 | 2.48 - 3.7 | 92 | 2.91 | 2.36 - 3.6 | 55 | 1.74 | 1.31 - 2.3 | 45 | 1.42 | 1.05 - 1.92 | 22 | 0.68 | 0.42 - 1.09 |
|  | Rawalpindi | 82 | 2.97 | 2.42 - 3.64 | 40 | 1.43 | 1.06 - 1.93 | 120 | 4.32 | 3.62 - 5.16 | 26 | 0.94 | 0.66 - 1.33 | 149 | 5.37 | 4.59 - 6.28 | 83 | 2.98 | 2.44 - 3.65 |
|  | Sahiwal | 11 | 0.65 | 0.35 - 1.2 | 7 | 0.39 | 0.19 - 0.82 | 51 | 2.87 | 2.09 - 3.92 | 43 | 2.43 | 1.75 - 3.37 | 70 | 3.94 | 2.95 - 5.24 | 53 | 3.01 | 2.24 - 2.04 |
|  | Sargodha | 116 | 5.16 | 4.31 - 6.18 | 107 | 4.81 | 3.99 - 5.77 | 81 | 3.61 | 2.9 - 4.48 | 35 | 1.6 | 1.13 - 2.24 | 192 | 8.56 | 7.35 - 9.96 | 157 | 7.04 | 5.93 - 8.34 |
|  | **Provincial** | **685** | **2.5** | **2.3 - 2.71** | **517** | **1.89** | **1.72 - 2.08** | **895** | **3.26** | **3.03 - 3.51** | **412** | **1.5** | **1.36 - 1.68** | **1232** | **4.49** | **4.21 - 4.78** | **858** | **3.13** | **2.9 - 3.38** |
| **Sindh** | Hyderabad | 424 | 16.72 | 15.04 - 18.54 | 326 | 12.89 | 11.44 - 14.49 | 46 | 1.81 | 1.39 - 2.4 | 30 | 1.17 | 0.81 - 1.69 | 40 | 1.58 | 1.16 - 2.16 | 17 | 0.67 | 0.38 - 1.2 |
|  | Karachi | 417 | 9.16 | 7.85 - 10.67 | 281 | 6.18 | 5.07 - 7.52 | 76 | 1.68 | 1.25 - 2.25 | 10 | 0.22 | 0.1 - 0.48 | 106 | 2.33 | 1.7 - 3.18 | 50 | 1.09 | 0.65 - 1.85 |
|  | Larkana | 25 | 1.87 | 1.27 - 2.74 | 7 | 0.49 | 0.23 - 1.03 | 43 | 3.2 | 2.38 - 4.28 | 24 | 1.78 | 1.18 - 2.67 | 35 | 2.62 | 1.74 - 3.92 | 19 | 1.43 | 0.83 - 2.48 |
|  | Mirpur Khas | 46 | 4.63 | 3.33 - 6.41 | 36 | 3.63 | 2.5 - 5.24 | 7 | 0.71 | 0.33 - 1.5 | 5 | 0.5 | 0.2 - 1.23 | 15 | 1.52 | 0.74 - 3.08 | 8 | 0.78 | 0.26 - 2.31 |
|  | Shaheed Benazirabad | 48 | 4.08 | 2.62 - 6.29 | 30 | 2.55 | 1.45 - 4.42 | 33 | 2.76 | 1.86 - 4.07 | 24 | 2.02 | 1.27 - 3.2 | 13 | 1.13 | 0.63 - 2.01 | 0 | 0 | 0 |
|  | Sukkur | 35 | 2.8 | 1.79 - 4.36 | 19 | 1.5 | 0.89 - 2.5 | 47 | 3.76 | 2.31 - 6.06 | 23 | 1.89 | 1.1 - 3.21 | 14 | 1.14 | 0.5 - 2.57 | 5 | 0.39 | 0.14 - 1.02 |
|  | **Provincial** | **995** | **8.38** | **7.7 - 9.12** | **699** | **5.89** | **5.32 - 6.53** | **252** | **2.13** | **1.82 - 2.49** | **116** | **0.98** | **0.8 - 1.21** | **223** | **1.89** | **1.57 - 2.28** | **99** | **0.83** | **0.61 - 1.14** |
| **KP** | Bannu | 28 | 3 | 1.85 - 4.83 | 21 | 2.26 | 1.32 - 3.86 | 2 | 0.18 | 0.004 - 0.75 | 1 | 0.009 | 0.001 - 0.67 | 4 | 0.42 | 0.17 - 1.05 | 0 | 0 | 0 |
|  | D.I.Khan | 44 | 3.73 | 2.65 - 5.23 | 33 | 2.87 | 1.9 - 4.31 | 9 | 0.8 | 0.31 - 2.0 | 0 | 0 | 0 | 16 | 1.36 | 0.72 - 2.53 | 4 | 0.35 | 0.001 - 1.19 |
|  | Hazara | 80 | 2.99 | 2.3 - 3.88 | 42 | 1.56 | 1.08 - 2.24 | 29 | 1.09 | 0.76 - 1.57 | 2 | 0.006 | 0.001 - 0.3 | 21 | 0.77 | 0.42 - 1.41 | 2 | 0.008 | 0.003 - 0.23 |
|  | Kohat | 18 | 1.35 | 0.88 - 2.07 | 8 | 0.64 | 0.36 - 1.14 | 13 | 1 | 0.66 - 1.53 | 1 | 0.005 | 0.001 - 0.21 | 7 | 0.57 | 0.3 - 1.07 | 1 | 0.004 | 0.001 - 0.34 |
|  | Malakand | 17 | 0.47 | 0.3 - 0.73 | 3 | 0.007 | 0.003 - 0.17 | 22 | 0.62 | 0.42 - 0.92 | 2 | 0.004 | 0.001 - 0.16 | 10 | 0.27 | 0.15 - 0.5 | 1 | 0.002 | 0.001 - 0.2 |
|  | Mardan | 15 | 0.87 | 0.47 - 1.59 | 2 | 0.008 | 0.001 - 0.59 | 21 | 1.19 | 0.66 - 2.13 | 0 | 0 | 0 | 18 | 1.03 | 0.56 - 1.91 | 0 | 0 | 0 |
|  | Peshawar | 9 | 0.26 | 0.001 - 0.64 | 0 | 0 | 0 | 7 | 0.21 | 0.009 - 0.49 | 0 | 0 | 0 | 6 | 0.16 | 0.004 - 0.57 | 0 | 0 | 0 |
|  | **Provincial** | **211** | **1.41** | **1.2 - 1.67** | **109** | **0.73** | **0.58 - 0.92** | **103** | **0.7** | **0.56 - 0.87** | **6** | **0.04** | **0.001 - 0.06** | **82** | **0.55** | **0.41 - 0.72** | **8** | **0.05** | **0.002 - 0.11** |
| **Pooled** | **National** | **1891** | **3.49** | **3.26 - 3.73** | **1325** | **2.45** | **2.26 - 2.65** | **1250** | **2.31** | **2.16 - 2.47** | **534** | **0.99** | **0.9 - 1.09** | **1537** | **2.84** | **2.66 - 3.03** | **965** | **1.78** | **1.64 - 1.94** |

*KP = Khyber-Phaktunkhwa, ST = smokeless tobacco*

**Table S3**: Estimates of logistic regression analyses for current tobacco use (as an outcome) and socio-demographic characteristics (education, area of residence, wealth index and age) (as explanatory variables) among women of reproductive age (15-49 years). Current tobacco use was investigated for each type (smokeless tobacco use, cigarette smoking, and waterpipe smoking,) separately, reference group in each case was those that are not currently using that type of tobacco. Adjusted odds ratios are reported below.

|  | **Smokeless tobacco**  **(N = 144,244)** | | **Cigarette smoking**  **(N = 143,775)** | | **Waterpipe smoking**  **(N = 144,271)** | |
| --- | --- | --- | --- | --- | --- | --- |
|  | **OR (95% CI)** | **P-value** | **OR (95% CI)** | **P-value** | **OR (95% CI)** | **P-value** |
| **Education (ref = none)** | | | | | | |
| Primary | 0.5 (0.3-0.9) | 0.02 | 0.7 (0.3-1.5) | 0.4 | 1.1 (0.5-2.6) | 0.8 |
| Middle | 0.4 (0.2-0.8) | 0.00 | 0.4 (0.1-0.9) | 0.04 | 0.7 (0.2-2.2) | 0.6 |
| Secondary | 0.2 (0.1-0.5) | 0.00 | 0.2 (0.1-0.4) | 0.00 | 0.5 (0.2-1.5) | 0.2 |
| Higher | 0.1 (0.05-0.3) | 0.00 | 0.1 (0.0-0.6) | 0.01 | 0.2 (0.1-0.4) | 0.00 |
| **Residence (ref = urban)** | | | | | | |
| Rural | 0.47 (0.3-0.9) | 0.02 | 0.7 (03-1.4) | 0.3 | 1.1 (0.4-2.9) | 0.8 |
| **Wealth Index (ref = poorest)** |  |  |  |  |  |  |
| Second | 0.5(0.4-0.5) | 0.00 | 0.6 (0.5-0.7) | 0.00 | 0.5 (0.4-0.6) | 0.00 |
| Middle | 0.4 (0.3-0.6) | 0.00 | 0.3 (0.2-0.4) | 0.00 | 0.3 (0.2-0.4) | 0.00 |
| Fourth | 0.3 (0.2-0.5) | 0.00 | 0.2 (0.1-0.3) | 0.00 | 0.2 (0.1-0.7) | 0.00 |
| Richest | 0.2 (0.1-0.4) | 0.00 | 0.1 (0.04-0.2) | 0.00 | 0.1 (0.0.5-0.3) | 0.00 |
| **Age** | 1.04 (0.9-1.1) | 0.05 | 1.1 (1.0-1.11) | 0.00 | 1.07 (1.05-1.1) | 0.00 |

*Note: The absolute numbers of women who used smokeless tobacco were 2530, smoked cigarettes were 775, and smoked waterpipe were 1791.*

**Table S4**: Sociodemographic distribution (weighted) of women with a live birth in the past 2 years based on the birth weight data.

| **Sociodemographic characteristics of women who had a live birth in the past 2 years based on birth weight data** | | | | | | | | | | | | | |
| --- | --- | --- | --- | --- | --- | --- | --- | --- | --- | --- | --- | --- | --- |
| **Province** | **Women (weighted)** | **Mean Age (years)** | **Rural dwellers  n (%)** | **Education  n (%)** | | | | | **Combined Wealth Index n (%)** | | | | |
|  |  |  |  | **None/preschool** | **Primary** | **Middle** | **Secondary** | **Higher** | **Poorest** | **Second** | **Middle** | **Fourth** | **Richest** |
| **Birth weight data based on delivery records** | | | | | | | | | | | | | |
| **Punjab** | 264 | 28.94 | 98 (33.3) | 37 (12.6) | 37 (12.6) | 32 (10.9) | 63 (21.4) | 125 (42.5) | 7 (2.3) | 19 (6.5) | 52 (14.1) | 68 (23.2) | 159 (54.1) |
| **Sindh** | 279 | 28.2 | 55 (18.7) | 106 (36.3) | 25 (8.6) | 21 (7.1) | 65 (22.2) | 76 (25.8) | 11 (3.9) | 26 (9.0) | 42 (14.3) | 93 (31.9) | 120 (40.9) |
| **KP** | 130 | 28.8 | 112 (77.9) | 47 (32.8) | 21 (14.3) | 15 (10.7) | 16 (10.9) | 45 (31.2) | 5 (3.3) | 20 (13.9) | 12 (8.4) | 37 (25.9) | 70 (48.6) |
| **Birth weight data based on recall** | | | | | | | | | | | | | |
| **Punjab** | 2261 | 28.88 | 1188 (50.9) | 394 (16.9) | 338 (14.5) | 276 (11.8) | 500 (21.4) | 828 (35.5) | 133 (5.7) | 239 (10.2) | 395 (16.9) | 577 (24.7) | 991 (42.4) |
| **Sindh** | 778 | 28.7 | 203 (23.5) | 252 (29.2) | 107 (12.5) | 101 (11.8) | 152 (17.7) | 248 (28.9) | 59 (6.8) | 78 (9.0) | 130 (15.1) | 265 (30.8) | 330 (38.3) |
| **KP** | 327 | 28.7 | 282 (80.7) | 117 (33.4) | 41 (11.9) | 38 (10.9) | 63 (18.1) | 89 (25.6) | 18 (5.1) | 26 (7.5) | 45 (13.1) | 88 (25.3) | 171 (49.0) |
| **Birth weight data not recorded** | | | | | | | | | | | | | |
| **Punjab** | **10480** | 28.52 | 7609 (72.6) | 5225 (49.9) | 2205 (21.0) | 1016 (9.7) | 1174 (11.2) | 860 (8.2) | 3036 (29.0) | 2428 (23.2) | 2151 (20.5) | 1782 (17.0) | 1083 (10.3) |
| **Sindh** | **4069** | 28.7 | 2802 (68.9) | 3087 (75.9) | 432 (10.6) | 146 (3.6) | 209 (5.1) | 195 (4.8) | 1281 (31.5) | 1237 (30.4) | 833 (20.5) | 464 (11.4) | 255 (6.3) |
| **KP** | **7712** | 28.3 | 6603 (85.6) | 5158 (66.9) | 898 (11.7) | 568 (7.4) | 559 (7.3) | 528 (6.9) | 1623 (21.0) | 1589 (20.6) | 1574 (20.4) | 1631 (21.1) | 1295 (16.8) |

*KP = Khyber-Phaktunkhwa*
